# Examining the Use of VR as a Study Aid for University Students with ADHD

**DOI:** 10.1145/3613904.3643021

**Published:** 2024-05-11

**Authors:** Isabelle Cuber, Juliana G. Goncalves De Souza, Irene Jacobs, Caroline Lowman, David Shepherd, Thomas Fritz, Joshua M. Langberg

**Affiliations:** University of Zurich, Switzerland; University of Zurich, Switzerland; Virginia Commonwealth University, United States; Virginia Commonwealth University, United States; Louisiana State University, United States; University of Zurich, Switzerland; Rutgers University, United States

## Abstract

Attention-deficit/hyperactivity disorder (ADHD) is a neurodevelopmental condition characterized by patterns of inattention and impulsivity, which lead to difficulties maintaining concentration and motivation while completing academic tasks. University settings, characterized by a high student-to-staff ratio, make treatments relying on human monitoring challenging. One potential replacement is Virtual Reality (VR) technology, which has shown potential to enhance learning outcomes and promote flow experience. In this study, we investigate the usage of VR with 27 university students with ADHD in an effort to improve their performance in ctableompleting homework, including an exploration of automated feedback via a technology probe. Quantitative results show significant increases in concentration, motivation, and effort levels during these VR sessions and qualitative data offers insight into considerations like comfort and deployment. Together, the results suggest that VR can be a valuable tool in leveling the playing field for university students with ADHD.

## 1 INTRODUCTION

Attention-Deficit/Hyperactivity Disorder (ADHD) is a highly prevalent neurodevelopmental condition, characterized by significant difficulties sustaining attention [6, 63]. Individuals with ADHD, despite having intellectual capabilities within the normal range, struggle with focus and distractibility and frequently make careless mistakes in academic work [5, 11, 35, 54, 60]. Furthermore, individuals with ADHD often exhibit deficits in academic motivation, particularly when working on tasks that require sustained mental effort [55, 66, 68]. Difficulties with attention and motivation can lead students with ADHD to take two or three times longer to complete tasks compared to their peers, or to avoid academic tasks altogether [27]. Long-term, these challenges negatively impact academic achievement and lead to low and failing grades and school dropout [5, 42, 65].

Evidence-based treatments for ADHD include medication and behavioral interventions. These treatments are effective but have significant drawbacks for use with emerging adults [16, 74]. For instance, while medication has a significant positive impact on symptoms of ADHD, many adolescents and emerging adults (up to 60%) who take medication in childhood, stop taking medication in adolescence [2]. Perhaps because of this, ADHD medication has a small and limited long-term impact on improving academic outcomes [41, 43]. An alternative to medication is behavioral treatment, which is effective at improving ADHD-related behaviors and some aspects of functioning. However, this type of treatment requires frequent and consistent monitoring from parents and/or teachers, which is time and resource-intensive and not feasible on a college campus [8, 19, 22, 36]. Due to the nature of a university—often characterized by a high student-to-staff ratio, informal study settings, and the absence of parental involvement—regularly scheduled, direct monitoring of students is not feasible [29]. Universities need feasible alternative support for college students with ADHD.

One promising way forward is to leverage Virtual Reality (VR) technology. This technology has made significant progress in recent years, unlocking potential use cases by addressing known limitations, such as improving screen resolution to make even small text readable and improving headset tracking to reduce motion sickness [58]. In many ways, VR is consistent with accommodations that universities provide for students with ADHD quite naturally. For instance, a common accommodation is to provide students with a structured, quiet environment while taking tests, to reduce distractions and improve focus and efficiency. Such an environment can be easily created in a virtual world, using a VR headset and noise-canceling headphones, without requiring a separate physical space. Further, because the digital environment makes it easy to monitor the student’s activity (e.g., using computer interaction monitoring, eye-tracking, etc.), it becomes feasible to automate the core principles of evidence-based behavioral treatment: consistent and frequent monitoring of attention linked with rewards for meeting goals.

To investigate the potential of using VR as an accommodation support for ADHD, we conducted a multi-week mixed methods study with 27 university students who met the DSM-5 criteria for ADHD [6]. Each student completed up to twelve 50-minute sessions in a VR environment and with noise-canceling headphones while working on their normal academic tasks. That is, students chose the academic homework and/or studying tasks that they would complete in VR each session and were not given any artificial tasks. After each session, participants completed questionnaires, and at the end of the study, in-depth interviews were conducted to gather insights about their VR experiences.

The primary goal of the study was to evaluate the impact of VR and noise-canceling headphones. However, we also wanted to explore the impact of providing students with feedback about their attention through a technology probe [34]. This probe, which used computer interaction data to provide users with positive or negative feedback, mimicked evidence-based ADHD treatment of continuous monitoring of students’ attention levels. The probe was piloted with the participants who took part in sessions 6 to 10.

The quantitative data analysis revealed a significant increase in concentration, efficiency and motivation levels during the VR study sessions compared to the baseline measures. Additionally, interviews conducted with participants yielded insights indicating that using VR technology and noise-canceling headphones helped mitigate external distractions and enhance work efficiency. Notably, participants exhibited a consistently high level of concentration across all study sessions, with no statistically significant differences observed between sessions in which they received feedback and those in which feedback was absent. This paper makes the following contributions:

- Empirical findings from a study with 27 university students with ADHD on the effects of VR on concentration, efficiency, and motivation while performing academic tasks regularly over a time span of several weeks.
- Empirical findings on the impact of using a technology probe for automated feedback based on participants’ computer interaction, also highlighting design considerations for such feedback mechanisms in VR.

Overall, this paper delves into the prospective advantages of VR technology as a tool for helping university students with ADHD, with a particular emphasis on feasibility and usability.

## 2 RELATED WORK

This work is necessarily cross-disciplinary, and so several areas of research are relevant. In this section, we first outline existing evidence for accommodation and behavioral support for ADHD. Then we discuss applications of VR technology for educational and work contexts. Finally, we summarize recent studies focused on the use of VR technology to support individuals with ADHD.

### 2.1 Academic Supports for University Students with ADHD

*Accommodations* represent adjustments to instructional practices and physical spaces to reduce the effects of a disability and improve demonstration of knowledge [31]. The empirical support for academic accommodations for students with ADHD is limited despite the frequency with which they are administered [64]. In a systematic review, Harrison et al. [33] found that of the 12 potential accommodations reviewed, only 4 were evaluated in more than one study, and only 5 of the 12 were evaluated with more than 10 participants across all studies. In a more recent review, Lovett and Nelson [46] identified 68 relevant studies, but concluded that the most common accommodations have few or no experimental studies directly investigating their efficacy. The limited research on setting accommodations designed to remove distractions while completing work is briefly reviewed here given the potential parallels to placing students in a distraction-free VR environment.

As with the overall accommodation literature, research on setting accommodations is less than conclusive. Vaughan et al. [73] found that although children with ADHD were more likely than typically developing peers to give invalid responses on memory and reaction time measures in a group setting, performance validity between groups was comparable when measures were administered individually. Smith and Riccomini [67] reported that students with learning disabilities in grades 3 to 5 demonstrated greater improvement in reading comprehension relative to their typically developing peers when wearing noise-reducing headphones. Contradicting this, Lin and Lin [45] found that students with learning disabilities did not benefit from a reduced noise environment when tested on number sense and numeration skills. Closest to our target population, Nelson and Lovett [49] administered two parallel forms of a timed silent reading comprehension test to a sample of college students with ADHD, one in a classroom with other students, and one in a private, proctored setting. A two-way analysis of variance found no significant main effects for either ADHD status or test setting on performance, and no significant interaction. Given the small sample for statistical tests (N = 27), the authors analyzed within group differences and found that 41% of students with ADHD diagnoses showed substantial benefit, and there were associations between severity of self-reported ADHD/distractibility symptoms and degree of benefit. However, the authors note that separate room accommodations present significant logistical difficulties for schools. Using VR technology could lower the logistical difficulties of providing an environment with reduced distractions to students with ADHD.

*Behavioral treatments* have been shown to improve the academic functioning of individuals with ADHD [5, 19, 22]. Specific behavior treatments, such as behavioral parent training or classroom contingency management, are well-established evidence-based treatments [22, 25]. They require parents and/or teachers to specifically define a behavior (e.g., attention) and to frequently and consistently monitor the behavior and provide feedback and rewards for use of the behavior or skill. These treatments have shown efficacy in randomized trials [3, 44], yet they require significant time and effort from mental health clinicians and parents to implement and suffer from significant feasibility challenges [29]. In addition, these treatments do not support students in the moment when completing academic work. In a university setting, where class sizes are larger, parent involvement is limited, and the demand to sustain focus throughout long time periods is greater [56, 57], supporting students with ADHD in the moment while completing academic work is simply not feasible.

### 2.2 VR for Education and Work

Several studies have evaluated the potential of VR technology for education and training in areas like medicine [62] or math [69], showing improved motivation and learning compared to traditional learning methods. In a recent study, participants played a serious game either in VR or with pen and paper. While there was no significant difference in learning between the two groups, the former reported higher levels of intrinsic motivation and flow [40], suggesting that VR can help foster flow in certain situations.

There is also a growing body of research on using VR for work-related tasks, fundamentally motivated by the prospect of offering an unconstrained virtual work environment, thereby circumventing the limitations and distractions intrinsic to traditional work settings [30]. While this research started in domain-specific endeavors, such as surgery [38] and 3D modeling [4], recent work has begun to investigate its use for office work in general. Some research in this area has focused on new forms of interacting with virtual content using VR and additional devices [13, 15], examined design aspects, challenges, and opportunities for a VR workplace [7, 30] and mobile virtual work [50], as well as needs for long-term immersion [32].

The most relevant research has, like the popular Job Simulator game^1^, asked participants to perform work-related tasks in a VR setting. For instance, Kiluk et al. had 15 participants engage in logic-based tasks simulating deep work in three different VR environments: a dimly lit room, a minimally adorned office, and a fully furnished apartment. Participants reported the highest relaxation in the minimalist office environment, with a heightened concentration in both the dimly lit room and minimally adorned office space [39]. Ruvimova et al. conducted a controlled lab study in which 35 participants were asked to perform visual programming tasks in four combinations of physical (open or closed office) and virtual (beach or virtual office) environments. Their findings indicate that participants in open offices were able to perform better when working in VR, as it reduced their distractions and increased their flow [61]. Pavanatto et al. conducted an experiment involving 18 participants, demonstrating that despite technical constraints, utilizing virtual monitors presented via Mixed Reality (MR) glasses represents a viable alternative to physical monitors when executing a productivity-oriented task (filling out forms) at a desk [53]. Going even further, Biener et al. [14] asked their participants to complete their real-world daily work tasks using a simple and cheap VR headset for eight hours a day, five days in a row. The results reveal major difficulties related to discomfort and usability but, at the same time, show some indications that participants were able to gradually overcome some of the initial problems.

These studies hint at the potential of VR environments, especially minimal office space, to help university students with ADHD block out the noisy dorm-room settings and concentrate on their work. Similar to Biener et al. [14], we focus on participants performing real-world tasks, such as homework, instead of games or specific and narrowly defined tasks. Different from prior work that focused primarily on cohorts comprised of knowledge workers or domain experts, we target university students with ADHD, a group that could particularly benefit from this support.

### 2.3 VR to Help Individuals with ADHD

To date, VR has not been applied to students with ADHD completing real-world academic tasks. In fact, most work in this space has used VR during assessment, not support, of ADHD. One approach embedded a continuous performance test (CPT) to assess ADHD symptoms and executive functions within a VR environment [51, 75]. The findings indicate that VR-based CPT offers advantages in comparison to conventional CPT methodologies, namely higher ecological validity [52] and a higher correct classification rate [1], as it provides absolute control over the testing environment. VR has also been utilized for ADHD cognitive training interventions [20], yielding some degree of improvement in inattentive symptoms [59]. Specifically, evidence suggests that youth with ADHD find virtual environments engaging and are better able to attend and progress with cognitive training [12]. However, completing actual homework and studying in VR is fundamentally different from completing structured assessments or cognitive training game-like tasks in VR. The previously mentioned Job Simulator game, where users complete simulated tasks such as entering three-character passwords, editing four-line spreadsheets, and making virtual coffee, illustrates the huge difference between simulated work in VR, and completing real academic tasks in VR. Challenges around supporting smaller text sizes, interacting with a physical keyboard while working in a virtual world, and many other unforeseen issues make it unclear if using VR is feasible and acceptable across a wide variety of academic tasks. Accordingly, our work focuses on understanding whether VR leads to significant improvements in concentration, effort, and motivation on real-world academic tasks and collects data on the user experience to ensure that VR is feasible and acceptable prior to scaling up the technology at universities.

## 3 METHODS

To investigate the use of VR technology for university students diagnosed with ADHD we focused on the following three research questions:

**RQ1** Can the use of VR increase concentration, motivation, and effort when working on academic tasks?
**RQ2** What is the impact and the potential for a longer-term use of VR?
**RQ3** Can automated feedback on users’ computer interaction within the VR foster attention?

To address the first two research questions, we performed a laboratory study with a cohort of 27 university students with ADHD. For the study, participants were asked to perform homework or studying activities within VR for up to twelve sessions, each lasting 50 minutes and with the sessions spanning several weeks.

To address RQ3 and explore whether we can mimic the evidence-based ADHD treatment of continuous monitoring of students’ attention levels, we incorporated ongoing feedback within the VR environment for up to 5 of the 12 sessions. Specifically, we developed an approach that monitors user’s computer interactions and conveys this information through a status bar integrated into the VR interface. While the interaction level only emulates a student’s attention levels, our approach served as a technological probe [34] to explore this feedback mechanism with students with ADHD.

Throughout the study and before, we collected quantitative and qualitative data, including data on participants’ concentration, effort, and motivation, participants’ interaction data when working in VR, and qualitative feedback from interviews with the participants. The questionnaires and interview questions can be found in the supplementary material [21]. The study was approved by our institutional ethics board prior to execution.

### 3.1 Participants

The study was advertised via a flyer distributed by the Student Accessibility and Educational Opportunity (SAEO) office, University Counseling Services, and Student Health. Using this flyer, we recruited 27 participants between the ages of 18 and 25 with a mean age of 21.04 years (±1.60). Out of 27 participants, 15 identified as female, five as male, five as non-binary, one as transgender, and one as genderfluid. Participants had to be at least 18 years old and attending the university where the study took place. They had to meet DSM-5 criteria for ADHD [6], endorsing at least six symptoms in the ADHD inattention domain as present and impairing during childhood and five symptoms as present and impairing currently. The consent form and IRB protocol specified that participants with a prior diagnosis of Autism or Bipolar and Obsessive Compulsive Disorder (OCD) were not eligible to participate. Given the high prevalence of anxiety and depressive disorders in emerging adults with ADHD, participants who reported prior or current diagnoses of anxiety or depression were eligible to participate.

Among the cohort of 27 participants, 23 met the criteria for ADHD Inattentive Presentation subtype, while 4 participants met the criteria for ADHD Combined Presentation. Seventeen participants were actively taking medication, and we instructed them to remain consistent with their current medication use throughout the study. As compensation, participants received $50 in the form of a gift card for completing the baseline assessment and another $50 for completing the post-intervention assessment. The study was approved by the university Institutional Review Board (IRB) and all participants signed informed consent.

### 3.2 Setup

**Figure 1:**
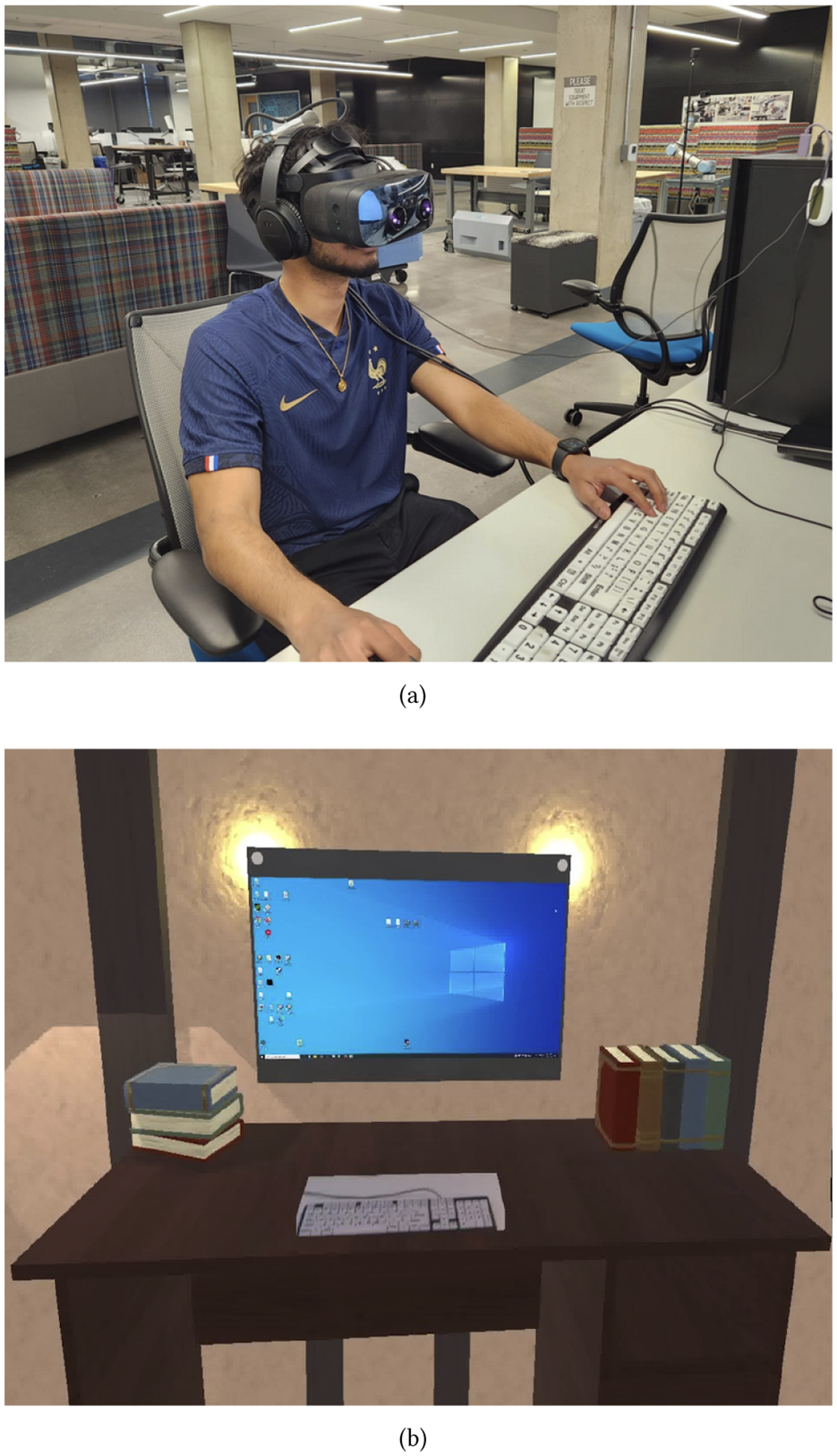
VR setup in an open university laboratory (a) and virtual cabin environment within the VR (b)

We used the Varjo XR-3 headset, a high-resolution industry headset (70ppd and 115-degree field of view), operated by a high-end graphics processing workstation (DigitalStorm workstation with Intel Core i9-10900K and GeForce RTX 3090 24GB graphics card), connected to a monitor, keyboard and mouse placed on a desk. While wearing the VR headset, participants were presented with a VR background showing the interior of a cabin with a desk in front of them as depicted in Fig. 1(b). The setting was designed with the objective of mitigating visual distractions and emulating a separate, quiet environment. The computer screen was projected in the center of the VR environment and was operated as a regular computer within the VR environment. The display was a mirror of a physical monitor, which had a resolution of 3840 × 2160, and in VR it was approximately the same size as the physical monitor, which was 28 inches. To facilitate typing, when participants looked down they could see their keyboard through a pass-through window. This allowed them to observe their fingers and hands in the physical environment, providing a direct view of their interactions with the keyboard and mouse. The displayed keyboard and mouse were not virtual but a direct reflection of their physical counter-parts, enabling participants to visually confirm their actions, such as key presses, in the real world. To eliminate auditory distractions, participants wore noise-canceling headphones. Driven by preceding findings highlighting the advantages of incorporating natural sounds as auditory stimuli [47], participants had the option to listen to ambient nature sounds during the VR sessions. The study setup was placed in an open office laboratory at a university, cohabited by both students and faculty members engaging in their respective tasks, shown in Fig. 1(a).

### 3.3 Procedure

#### 3.3.1 Screening and Baseline.

During the initial screening process, the Barkley Adult ADHD Rating Scale-IV (BAARS) [10] and the Barkley Deficits in Executive Functioning Scale (BDEFS) [37] were administered online to evaluate ADHD symptoms and related impairments. Participants also completed a demographics questionnaire and answered questions related to prior mental health diagnoses, epilepsy, seizure, and motion sickness to ascertain the presence of any medical issues that could compromise the safety and well-being of participants when engaging with VR technology.

Once participants passed the screening, they were asked to complete the questionnaires for the baseline evaluation assessing their typical levels of concentration, effort/efficiency, and motivation when completing homework and studying. Each of the three questionnaires contained seven items, using a four-point Likert scale. For the first scale, on concentration, participants responded to items that included how frequently they were (1) daydreaming, (2) lost in their own thoughts, (3) staring blankly into the space, (4) feeling like their mind was in a fog, and (5) inattentive. For the effort/efficiency scale, participants responded to statements such as, “I gave my best effort when completing the work”, “I was able to complete as many problems as I wanted to” and “I made the most of my time”. For motivation, statements such as “I felt motivated to complete the work” and “I felt motivated to give my best effort” were used. All items used in these three scales were taken from well-established assessment tools such as the Adult Concentration Inventory [26] and homework motivation scales [71, 72].

#### 3.3.2 VR Sessions.

Prior to coming to the laboratory for the first session, participants received an email containing instructions about the tasks they were expected to work on during the VR sessions. They were specifically asked to work on tasks that involved academic activities, such as homework or studying, which necessitated interaction with the computer. During the initial session, participants were introduced to the VR setup, and instructions regarding the study protocol were provided by the proctor. Additionally, any questions or concerns were addressed, and the proctor ensured that participants had suitable tasks to work on.

At the beginning of each session, the proctor worked with the participant to adjust the VR headset for comfort and clarity. Participants then worked on their tasks in VR for 50 minutes. This time period was chosen to be long enough to tax attention yet short enough to be realistic for individuals without attention difficulties to maintain focus on academic work. Participants could freely open any websites, including social media, and use all applications on the experiment computer or their phone. They were encouraged to ask questions, adjust the headset, or take a break whenever necessary. After the 50-minute work session, participants were asked to fill out the three questionnaires on concentration, motivation, and effort on the experiment computer. To get an understanding of how usable the VR system was and how comfortable participants felt using it, we further asked them to fill out the System Usability Scale [18] at the end of each session. Finally, the proctor asked a few short questions about the participant’s experience and whether and when they might have lost attention during the session.

Participants could book time slots that fit their schedule using an online calendar system set up by the experimenters. Analogous to established accommodations like testing or seating adjustments, we wanted to focus on the benefits in the moment instead of long-term outcomes or generalizations to other settings. To mitigate potential novelty effects associated with using VR glasses and examine the potential for longer-term use of VR, participants could take part in up to twelve experimental sessions, typically involving two sessions per week. The automated feedback was explored with a subset of participants who chose to keep using VR and participate in sessions 6 through 10.

#### 3.3.3 Automated Monitoring and Feedback (sessions 6 to 10).

To mimic continuous attention monitoring of students—an evidence-based behavioral ADHD treatment—we implemented an approach to provide ongoing feedback on their interaction within the VR system. During all sessions, the approach automatically gathered information on the interaction between the participant and the experiment computer. Specifically, the number of mouse clicks, the number of keystrokes, the scrolling distance, and cursor movement were logged and an input level that combined all four interaction types was calculated. For the calculation, we weighted each interaction type proportionally, accounting for the variation in measurement units: mouse and keyboard interactions are quantified by the number of clicks, while scrolling and mouse movement are expressed in pixels. We determined these weights in several pilots prior to the study, and the same weights were used for all participants.^2^ For each user, the approach calculated four thresholds (i.e., high, highest, low, and lowest) for input levels by adding or subtracting one-fourth or one-half of the standard deviation of the user’s average input level. In contrast to the weights, the thresholds were calculated for each participant individually, based only on their own interaction data, as we observed the amount of interaction to vary strongly between participants.

**Figure 2:**
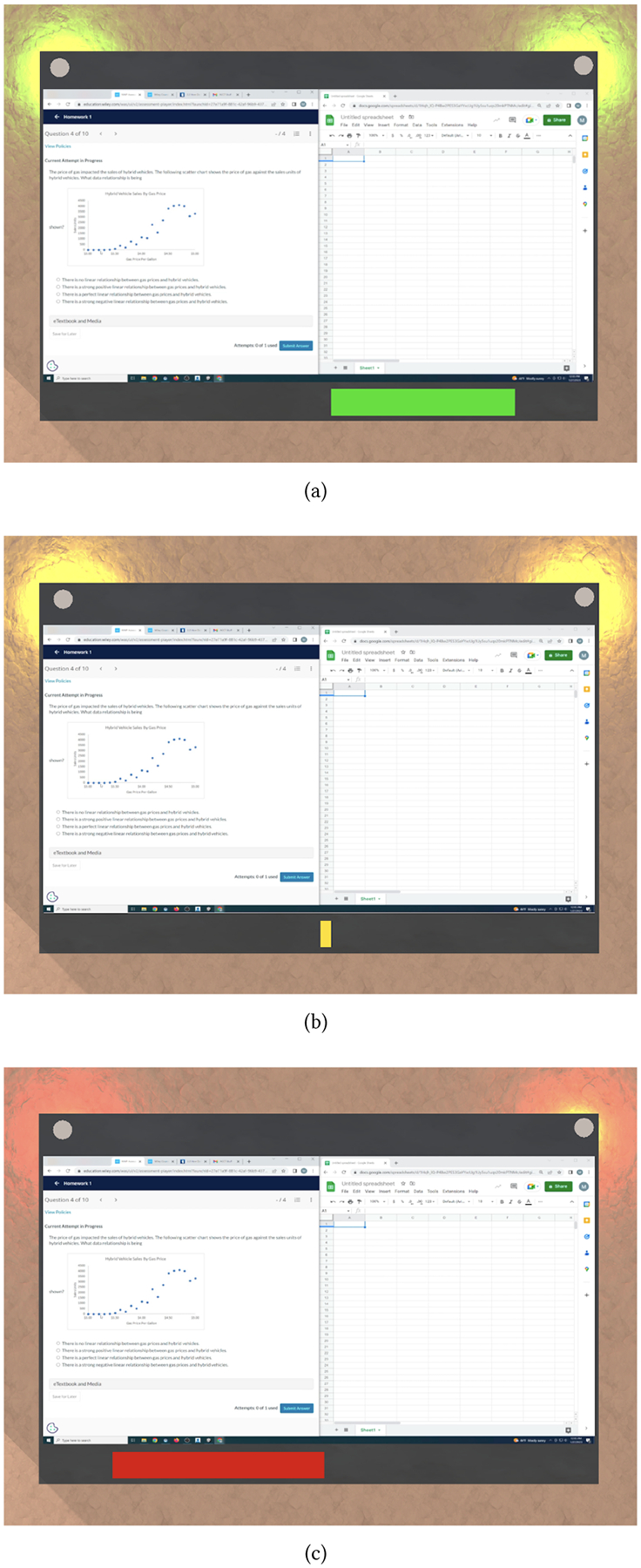
Monitor projected into the VR environment with a green (a), yellow (b), and red (c) feedback bar.

At intervals of one minute, the approach used all user interactions from the previous 60 seconds to calculate an input level. Based on the input level, the approach updated a feedback bar displayed in the VR environment, positioned directly underneath the monitor (see Figure 2). The feedback bar began as a small yellow bar in the center of the screen (shown in Figure 2(b)). Each minute, the approach either increased or decreased the bar based on the input level. That is, if the input level exceeded the high or highest threshold the bar increased by 5% or 10%, respectively. If the input level was less than the low or lowest threshold the bar decreased by 5% or 10%, respectively. However, if the input level fell between the low and high thresholds, instead of staying stagnant, the bar increased by 2.5%, as a way of encouraging users. To reinforce the feedback, the bar changed color as it grew or shrunk. Figure 2 depicts the monitor in the VR environment with the feedback bar in green (a), yellow (b), and red (c). We piloted this approach and adapted the thresholds and in/decrements to ensure that the bar was not too distracting.

We deployed our feedback approach as a technology probe with an ABA design in mind. We provided no feedback during sessions 1 to 5 and 11 to 12, only turning on the feedback for sessions 6 to 10. This also made it easier for participants to compare the two conditions.

#### 3.3.4 Post-Study Qestionnaire and Interview.

Upon the conclusion of 12 study sessions participants were requested to complete a follow-up assessment using the Barkley Adult ADHD Rating Scale-IV [10]. Note that some participants discontinued the study prior to twelve sessions, and were thus provided this assessment then. We contacted all participants after the study’s completion inviting them to additionally engage in a semi-structured final interview, which lasted approximately 45 minutes. The interview focused on users’ overall experience using the VR system, the utilized VR environment, the accuracy and usefulness of the feedback bar as well as potential future use. The interview questions and questionnaires can be found in the supplementary material [21].

### 3.4 Data Collection and Analysis

Participants could partake in a maximum of twelve VR sessions, distributed across multiple weeks, typically at the rate of two sessions per week. On average, participants completed 7.4 sessions, leading to 198 proctored sessions in total. Due to a software bug, interaction data was lost for 10 of the 198 sessions.

#### 3.4.1 Qestionnaires.

We calculated the concentration, motivation, effort, and usability scores using the respective questionnaire guidelines [18, 26, 71, 72]. The concentration, motivation, and effort scores were between 0 and 21, and the usability scores were between 0 and 100. For effort, motivation, and usability, higher numerical values signify superior performance. In the case of concentration, a lower numerical value corresponds to heightened concentration levels.

To compare between various groups of data (e.g., baseline data to VR session data, data from sessions with feedback to data from sessions without feedback, etc.), we used a series of statistical assessments. First, we assessed normality using the Shapiro-Wilk test and homogeneity of variances using Levene’s test. If none of the p-values from these tests were found to be statistically significant, we conducted paired t-tests. In instances where the Levene’s test yielded a p-value of less than 0.05, Welsh’s t-tests were applied to the respective dataset. For scenarios where only one or no side of the data exhibited a normal distribution, the Wilcoxon signed-rank test was employed. An independent t-test was executed to compare the average scores of participants who completed all 12 sessions to those who participated in fewer sessions. In any cases where we observed a difference, we measured effect sizes using Cohen’s d.

For the statistical analysis comparing VR and baseline condition, all participants who had filled out baseline and session 1 questionnaires were included (N=27). Conversely, for the statistical comparison between feedback and no feedback sessions, we included all participants who finished at least two feedback sessions (N=16).

#### 3.4.2 Interaction Data.

During all VR sessions, interaction data between the participant and experiment computer was tracked automatically. Specifically, the number of mouse clicks, the number of keystrokes, the scrolling distance, and the cursor movement distance were logged once every 10 seconds. The software utilized for data tracking was a modified version of PersonalAnalytics [48]. This data was the basis for the feedback participants received during some of the sessions to answer RQ3.

#### 3.4.3 Interviews.

We contacted all participants after the study’s completion inviting them to additionally engage in a semi-structured final interview. 17 of the 27 participants took part in a final interview. Interviews lasted between 19 and 51 minutes. The mean number of VR sessions attended by these 17 participants was 7.9, which was similar to the overall participant cohort’s mean session attendance of 7.4. Utilizing transcripts from the final interviews, we performed a thematic analysis [17]. Initially, three authors independently coded statements from four participants, identifying emerging themes, and engaged in discussions to refine them. Following this, one author systematically coded statements from the remaining 13 interviews using the established themes.

## 4 RESULTS

In this section, we report our findings showing the effects of university students using VR to complete academic work on their concentration, motivation, and effort levels. Next, we discuss participants’ experiences with VR usage over multiple sessions and weeks, offering insights into related issues such as headset comfort, customization preferences, and potential approaches for wider deployments. Finally, we present our findings related to the value of our automated interaction feedback.

### 4.1 Increased Concentration, Motivation and Effort

#### 4.1.1 Increased Concentration.

**Figure 3:**
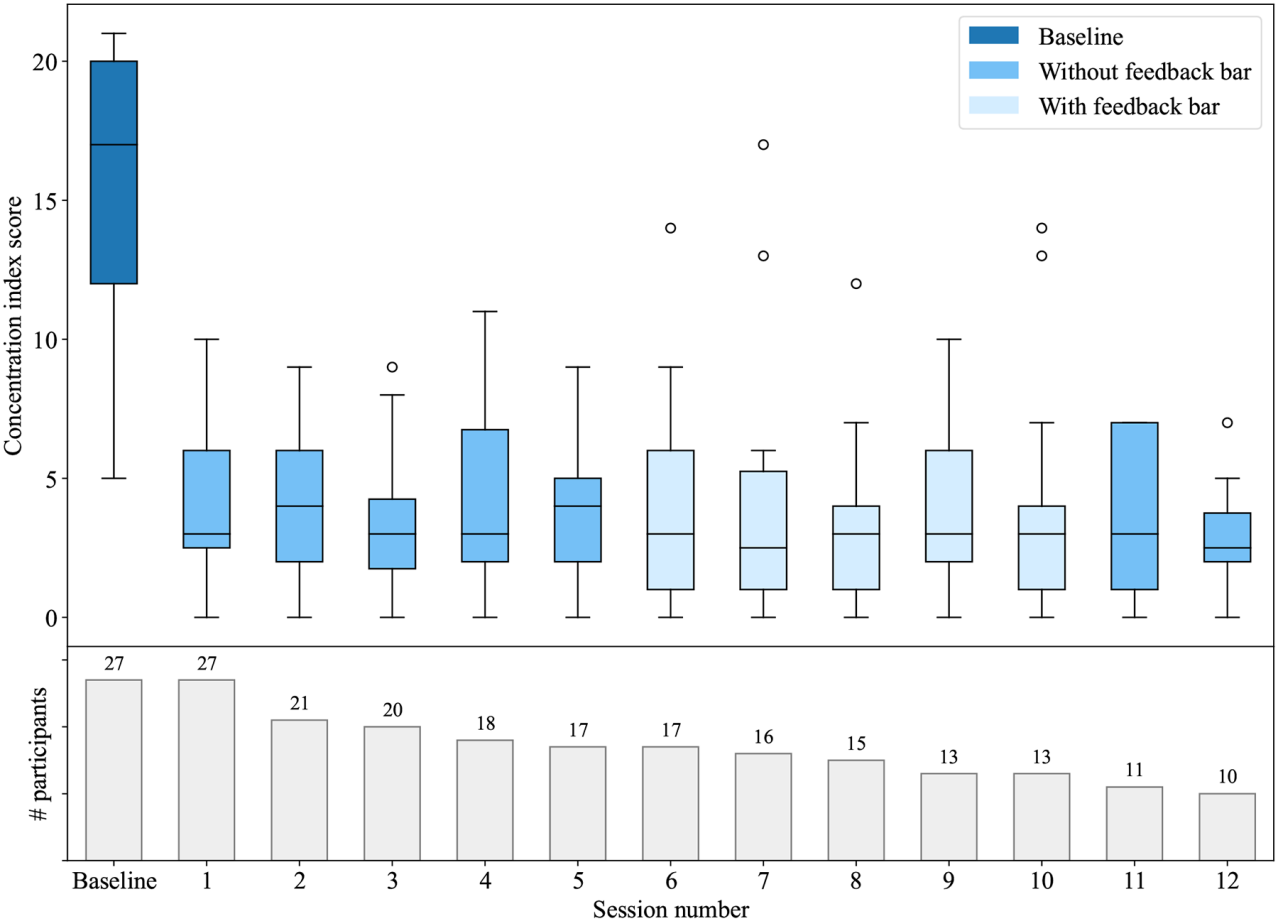
Concentration score per session (above), including baseline, with number of participants per session (below).

Overall, the VR environment had a significant effect on students’ concentration when comparing the baseline with the VR sessions, a finding that was supported by both qualitative (see Section 4.1.4) and quantitative data. Quantitatively, students’ concentration index scores for the VR sessions were markedly lower—indicating higher levels of concentration—compared to the students’ baseline concentration scores collected before the VR sessions. Figure 3 plots these scores by session and illustrates the significant difference between the baseline scores, shown on the left, and the scores for the VR sessions. Note that the VR sessions in which feedback was provided (light blue, sessions 6 to 10) had similar mean scores as the sessions without feedback. Table 1 presents the mean concentration scores together with the motivation and effort scores.

A statistical analysis of the data comparing the baseline and the average concentration index score across all VR sessions of a participant using Welsh’s t-test showed a significant difference (n=27, t(26)=11.17, p<0.0001) with large effect size (Cohen’s d=3.04). Similarly, a Welsh’s t-test between the concentration index score for the baseline and the first VR session showed a significant effect (n=27, t(26)=10.66, p<0.0001) with a large effect size (Cohen’s d=2.90).

#### 4.1.2 Increased Efficiency.

The VR environment also significantly enhanced students’ work efficiency. Quantitatively, this efficiency gain was substantiated by an increase in the effort index (see Table 1). Both, a paired t-test comparing the baseline to the average VR effort index score showed a significant increase (n=27, t(26)=−7.92, p<0.0001) with large effect size (Cohen’s d=1.76), as well as a paired t-test comparing the baseline to the first VR session (n=27, t(26)=−6.73, p<0.0001, Cohen’s d=1.54). In the interviews, participants further supported this increase in efficiency, stating that they were able to complete tasks more quickly and effectively within the VR environment. For example, P4 stated *“I would say I was able to use VR in a way I didn’t know was possible and to complete my assignments in a quicker and more efficient way. It helped me get things done.”*, and P13 mentioned *“It was a good way for me to stay focused and I completed like a lot more work than I thought I would be able to in an hour while using the VR”*.

#### 4.1.3 Increased Motivation.

Finally, participants reported experiencing significantly elevated motivation levels in the VR environment. The statistical analysis of the Homework Motivation Index data [71] revealed a significant increase with large effect Wilcoxon signed-rank test between the baseline and the first VR session (n=27, t(26)=−5.46, p=0.0001, Cohen’s d=1.25). For the latter, we used a Wilcoxon signed-rank test since the data was not normally distributed.

#### 4.1.4 Contributing Factors.

The increase in concentration, motivation, and effort was consistent, significant, and with a large effect size. To understand these changes more deeply we analyzed themes from the final interviews of participants. Although it is difficult to directly map between these themes and our quantitative findings, many of these themes provide *potential reasons* why the measures improved.

The higher concentration index score, which represents an increase in focus and attention, was also supported by participants’ statements in the interviews. For example, participant P6 stated *“for me it helped me lock in and stay focused”*. One of the contributing factors to this increase in focus was the reduction in distractions. Without being prompted, 15 of the 17 interviewed participants explicitly stated that the **VR environment together with the noise-canceling headphones reduced both visual and auditory distractions**, helping them to focus. Participant P15 remarked that *“It helped a lot in removing all distractions because that’s my main issue when getting my homework done. Like seeing people move, seeing other things happen, my brain always picks that up, so I always have to lift my head and look at that”*.

The participants also confirmed the importance of blocking out sound, which reduced distractions, potentially affecting measures such as concentration and efficiency. P2 said *“I have ADHD and autism and I fear everything that is going on around me, and sometimes I can hyper-fixate and block everything around me but I don’t have much control over it personally, it’s just like my brain is blocking it out, and if somebody is drinking water or coughing too much and it’s making me overstimulated”*. In these cases, the headphones and background audio used in our experiment can help reduce distractions. As P5 said: *“I think the noise-canceling headphones were really helpful. I also think… having the background noise be very plain and muffling out the actual background noise. The generated background noise like the birds and whatever, was really nice”*.

In addition to the external distractions that reduce focus, 6 participants without having been prompted for it also identified a **decrease in self-interruptions while working in VR**. Although access to neither phone nor social media was restricted during the experiment, the VR environment made access to both less convenient, as participants noted during the interviews: *“Because one of the main things is that VR, when you’re in that space, is a barrier between you and environmental distractions, but also personal distractions. When I was in the VR headset, I couldn’t check my phone; it would have been really hard to look through the camera to do that. I didn’t like open my messaging app or different stuff like that. So I think if you kind of honor the VR space and you don’t let fun things into it or like distractions into it, then it’s kind of reinforcing the study time: ‘We are not gonna look at Twitter, or Facebook, or watch a YouTube video.”‘* (P1). Similarly, participant P2 stated that *“the best thing about like being in this kind of world was that I could not see my phone, because I would need to like open the goggles up to see my phone, so I just didn’t.”* The sensory deprivation of the VR environment also fostered a *“tunnel vision”* (P7) and a focus on the planned work: *“I was only focused on the work. There was nothing for me on the desk to play with. There was like nothing for me to do except for work that was on the screen”* (P17). One participant even went as far as stating that the VR can serve as an overseer that helps them to stay focused on their tasks: *“The VR helmet is like my babysitter that helps me make sure I am doing it”* (P1).

Independent of the VR, 7 of the 17 participants also mentioned that the **regular, scheduled appointments for the VR sessions contributed to the increased concentration, effort, and motivation**. For example, participant P1 stated *“I thought it was really nice because my ADHD is mostly affected by external things like having deadlines or appointments, so scheduling an appointment where I have to go in and work on something for an hour made me feel a lot more productive.”* At the same time, all 7 participants who mentioned the impact of scheduled appointments also named the VR environment and the noise-canceling headphones as big factors in the improved focus. When asked about what went well for them during the VR sessions, participant P4, for example, answered: *“Definitely dedicating time out of my day to do it and the consistent schedule to do work. Also, the sensory deprivation aspect, my ADHD is worse with distractions, I was able to stay more focused”*. Participants even went as far as trying to recreate the experience outside the sessions and without a VR: *“I think that going to the VR room helped me get in the zone and I would try to recreate that. I would go to the library and put on bird noises to get in the zone to trick my brain that I am in that space.”* (P9).

#### 4.1.5 VR Environment.

Participants (15 of 17) agreed that the VR environment was well-suited for their academic tasks, yet desired some customization, especially for the audio. For all VR sessions, participants were placed in a simple cabin room (see Figure 1(b)) and they generally appreciated it: *“I think it was really nice and I liked it. I did actually like the low graphics; it felt like that made it less of a distraction. [..] The only other thing I can think of is like a library environment or a nature scene, but that might be too cool, and I would end up looking at it.”* (P1).

Yet, opinions regarding the accompanying nature audio varied. Several participants expressed a fondness for it, with two of them stating without having been prompted that they had begun using similar ambient sounds during their personal study routines post-study. However, for certain participants, the audio was perceived as distracting in specific instances, and they preferred alternative, simpler sounds like pink or white noise. For example, P8 stated *“I did like the birds, but I would hear bugs, but that was weird and bugs are distractions.”* and P11 said that they *“did not like the chirping birds. I would prefer hard rain sounds [..] drizzling, [and] thunderstorms”*. Without exception, all participants expressed a desire for customization features within the VR environment and its audio, including a beach or a library, and emphasized the wish to choose it freely to adjust to their preferences and habits: *“Typically when I study I listen to white noise or brown noise and I feel like something like that for a fellow someone who has pretty severe ADHD, that is the one noise to always help me out when focusing [..] I usually always have white noise or brown noise and I didn’t have it”* (P15).

### 4.2 Long-Term Use of VR

#### 4.2.1 Consistent and Continuous Positive Effect of VR.

The concentration level remained consistently high across all sessions, varying little between sessions even though these sessions spanned several weeks for most participants (see also Figure 3). The motivation and effort scores exhibit a similar consistency across participants’ VR sessions. A repeated-measures ANOVA using the twelve VR sessions as a within-participants factor revealed no significant difference between the sessions, neither in concentration (F(11,99)=0.46, p>0.92), motivation (F(11,99)=1.37, p>0.19) nor effort (F(11,99)=1.54, p>0.12).

This consistency across the 12 sessions and multiple weeks indicates that the positive effects of the VR environment for users with ADHD continue over longer periods of time and are not just a novelty effect or wear off quickly. Several participants further corroborated this in their interviews, stating that they would be happy to continue using the VR environment in the future and were even looking forward to more: *“Honestly, overall I found myself very much looking forward to each time I got to go [..] I wish there was more than the 12 sessions.”* (P8). Only one interview participant mentioned a novelty effect, yet also stated that the VR environment was effective nevertheless: *“I do think over time when the sense of novelty wore off it was less effective for me specifically, in that space. But because there was a history of me focusing in the space, body doubling, and routine I was still able to get things done”* (P9).

While more than half of the participants completed less than twelve VR sessions (see bottom of Figure 3), almost all of the ones that also participated in an interview mentioned reasons unrelated to the VR or its effects. Reasons for discontinuation included the end of the academic semester (3x), instances of illness and injury rendering laboratory attendance infeasible (2x), excessive work commitments (2x), and other personal reasons (2x). Only one participant explicitly expressed their disapproval of VR usage and consequently opted to discontinue their participation in the study. Statistical analysis in the form of independent t-tests did not show any significant differences in average scores between participants who finished 12 sessions and those who participated in a lesser number of sessions, neither in concentration (*n*_1_=10, *n*_2_=17, t(25)=−0.12, p=0.9041, Cohen’s d=0.05), effort (*n*_1_=10, *n*_2_=17, t(25)=0.75, p=0.4616, Cohen’s d=0.30), motivation (*n*_1_=10, *n*_2_=17, t(25)=−1.44, p=0.1617, Cohen’s d=0.54) nor usability (*n*_1_=10, *n*_2_=17, t(25)=1.01, p=0.3237, Cohen’s d=0.40).

#### 4.2.2 VR Well-Suited for Academic Tasks, in Particular Demanding Tasks.

Throughout the experiment, participants worked on a variety of tasks within the VR environment, ranging from reading, preparing presentations, and writing essays to completing online quizzes. Participants generally stated that the VR environment was well-suited for their tasks. Many participants further elaborated that the VR environment is particularly suitable for tasks that are demanding, *“require more brain power and effort”* (P4), are undesired, and during which they easily get distracted. For example, P1 stated *“Usually I brought philosophy to the [VR] sessions, which is my least favorite subject. It’s tedious, hard to read, so being in a space where I had to work on it was really motivating for me.”* and P13 mentioned that *“when I read that’s the most time when I end up getting distracted over something; so [the VR] helped a lot”*. Participants also mentioned that the VR helped them to start new assignments, which can often be difficult for those with ADHD.

At the same time, participants mentioned that the physical limitations of the VR can pose challenges for certain tasks, such as solving mathematical equations, referencing physical textbooks, or drawing on a tablet: *“I couldn’t do physics that much because you need a calculator and need to write it out”* (P14). Some participants therefore just planned the tasks they wanted to work on in the sessions.

#### 4.2.3 Ideal Duration of Sessions Varies by Participant and Task.

Overall, the ideal duration for a VR session varies by participant. A large number of participants (8 of 17) perceived the session duration of 50 minutes in our study to strike a good balance, as it provided enough time for meaningful progress without becoming overly long and exhausting: *“I think it was a great amount of time. I think it was enough time to get me a sufficient amount of work done but also didn’t get too tiring”* (P3). Other participants (3) would prefer longer sessions to complete their tasks *“I actually thought it was too short, almost every single time when the researcher would tell me ‘we’re done’ I would be like ‘oh, I just started’.”* (P15), and for some, the ideal duration varied by session and task: *“Sometimes it felt longer than others but sometimes it felt like almost too short of time to like finish something. But sometimes I was actually able to go through something and finish an assignment.”* (P12). Finally, for some participants (3 of 17) the 50-minute sessions in the VR were too long due to factors related to the discomfort of the VR equipment: *“30 minutes would probably be better. 50 minutes were too much, my eyes were tired”* (P5).

#### 4.2.4 VR Can Be Uncomfortable and Take a Few Sessions to Get Used To.

Despite some discomfort frequently associated with wearing the VR headset, all but one participant continued the VR sessions. The one participant who stopped reported motion sickness and withdrew from the study early on. In the interviews, participants mentioned discomfort due to eye strain (8x), headset weight (8x), headaches (4x), and calibration issues (2x). For example, P15 stated that *“it was heavy [..] slightly uncomfortable”*, and P17 talked about *“eye strain from it”* and that *“the headset [..] would tend to put a lot of pressure on my glasses’ frame”* since he was wearing glasses inside the VR. At the same time, some participants mentioned that it just needs some time to get used to it: *“the headset was pretty comfortable. You just have to make sure that it’s adjusted correctly, because otherwise if it’s too loose it will make you weigh down.”* (12).

Several participants mentioned that it takes some time to get used to the VR headset as well as the environment, yet it varied how long this took with a minimum of one and up to six sessions. For some *“it was pretty simple, as long as you know how to use a computer and how to navigate it, you can do it.”* (P2). On average, participants said they felt comfortable using the setup after around session 3: *“[..] for the first few sessions you’re still getting used to the system. Like by the end I really got to know the system and I got to know its quirks and what to look for and how to do certain things.”* (P17). Based on the system’s usability score, participants rated the system’s usability as good [9], with an average score of 72.71 ± 17.71, where a score above 68 is considered above average. The usability score also did not change significantly between each participant’s first and last session (mean first session 68.5±15.07, mean last session 73.5±20.72, n=21, paired t-test p>0.10, t(20)=−1.73, Cohen’s d=0.28).

#### 4.2.5 VR Sessions Better in the Library than at Home.

For the VR sessions in our study, we had participants come into a lab (see Figure 1(a)). While this setting was effective, we interviewed participants about other usage contexts, specifically the university library or at home, for a potential long-term solution. Overall, most participants preferred using the VR in a university library setting over using it at home. When we described the potential placement of the VR system within the university library, where it could be booked and used just like any other study room, 13 out of 17 participants indicated a strong interest: *“Oh I would love that, 100% yes”* (P2). Using the VR at home for work sessions was less appealing to participants and only 7 of 17 expressed interest. While the participants saw the benefits of using the VR for their work sessions, they were worried about other distractions and a lack of focus when using it at home. For example, P6 said that *“I’m too relaxed at home. If I want to be super focused I’ll go to a library or something”* and P8 weighed in on the differences between using the VR in the library and at home: *“I would benefit more from a library, yes, but it would depend on the environment. For me going somewhere to do something is more effective. Home brings a lot more opportunities for distraction.”* P16 also mentioned that using the VR at home might lead to spending too much time at home and *“getting dissociated with reality in a sense”*.

In addition, we asked participants about using VR for in-school tests and exams. While 6 participants endorsed the idea, 8 participants remained undecided. The perceived benefits included reduced external distractions and better focus; however, concerns centered on potential technological issues, heightened anxiety, and the possibility of creating unfair advantages for students with access to VR headsets.

### 4.3 Interaction Feedback

**Figure 4:**
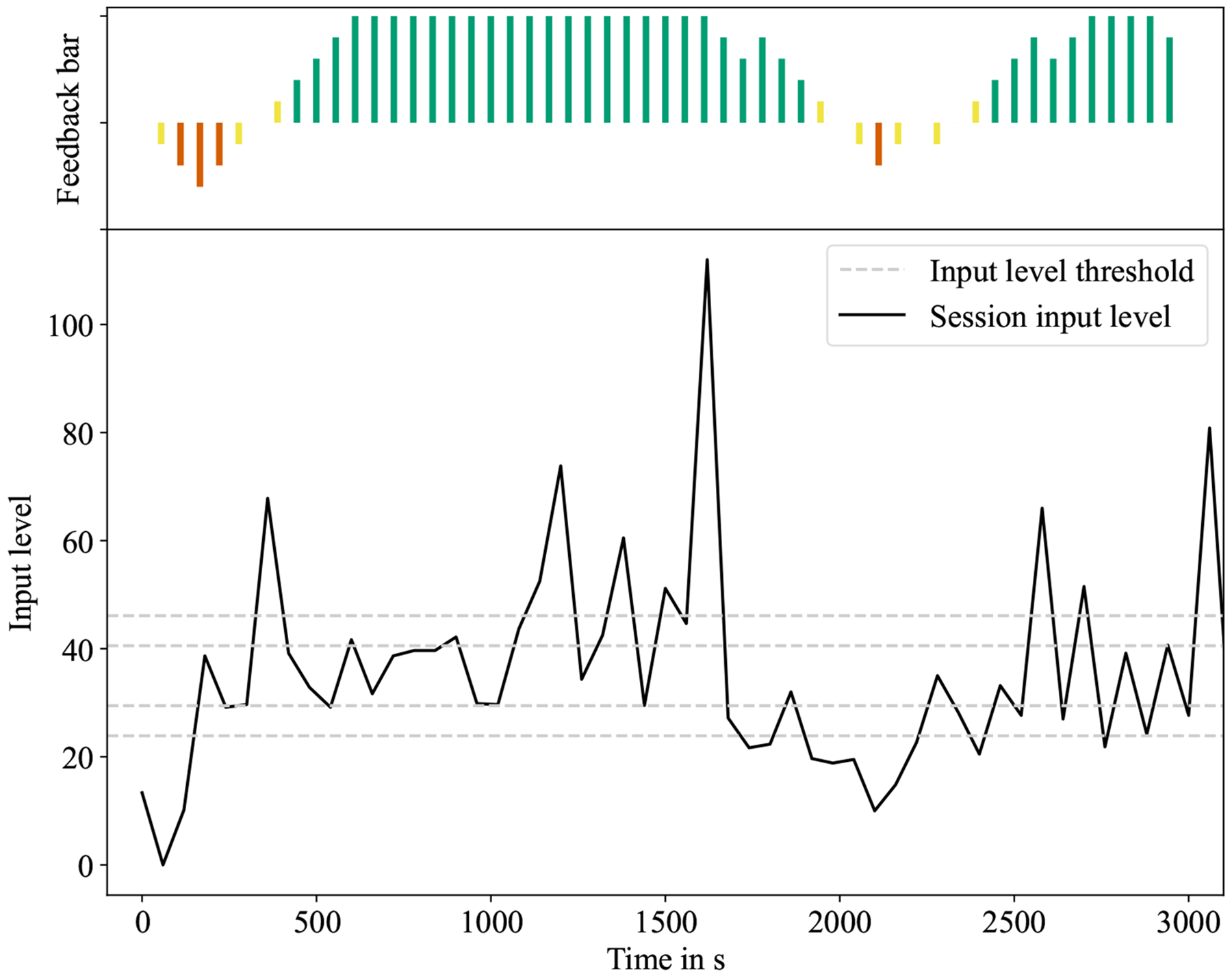
Visualization of a user’s feedback bar (top) and input level (bottom) over a single session. Note that dashed lines represent previously calculated thresholds (bottom).

Overall, the automated feedback on students’ interaction level that we deployed for sessions 6 to 10 had no statistically significant impact on motivation, effort, or concentration. Figure 4 depicts the calculated input level and feedback bar (size and color) over the course of one 50-minute session for one of the participants. Across all sessions and participants during which the feedback bar was displayed, it was green 69% of the time, yellow 6%, and red 25% of the time.

Performing paired t-tests between the average scores of a participant for sessions with and the ones without feedback revealed no statistically significant difference for concentration (n=16, t(15)=−0.24, p=0.815, Cohen’s d=0.04), effort (n=16, t(15)=0.05, p=0.960, Cohen’s d=0.01), or motivation (n=16, t(15)=0.18, p=0.859, Cohen’s d=0.03). For completeness, we also analyzed the usability scores and found no significant difference between the different types of sessions (n=16, t(15)=−0.51, p=0.617, Cohen’s d=0.05). For these tests, we included the 16 participants who completed at least two sessions with feedback and five without it.

Participants had differing views on the feedback bar, with some finding it valuable, while others perceived it as having limited value or even causing distraction. In the interviews with 12 participants who experienced at least one session with the feedback bar, four participants expressed positivity, finding the bar to be motivating, helpful, and fostering them to work. P9 stated that the bar *“was very useful since I am very competitive. It helped me lock onto whichever task I was doing. The bar would help me start the hyperfocus and be locked in on the environment. [..] I really liked it because I was in competition with myself”* (P9). Participants also mentioned that they *“like how the color changed”* (P4) and P12 pointed out the encouraging effect of it: *“Yeah, when it started turning it was very like noticeable versus the yellow that wasn’t like that. So when you see the red you are like “go back to work”. [..] I feel the feedback bar was the best thing for me.”*

On the other hand, several participants had more mixed or even negative feelings about the feedback bar. Participants indicated that they did not pay attention to it, e.g., *“I didn’t pay much attention since it didn’t change colors. It blended into the background.”* (P6), or only noticed and missed it once it was not there anymore *“I thought it was just kind of there because we started without it, and then we got it. In the end, they would ask me if it helped, and I always said it was just kind of there. But then they took it away, and then I missed it, and I was like, wow, maybe it was actually doing something for me. I looked, and the bar was not there; it was like my emotional support bar. I wasn’t told that I was doing good anymore.”* (P1). The bar also evoked emotions in participants such as anxiety and was *“a little intimidating”* (P5). Even though P17 thought that the bar was useful, they would prefer it not to *“turn red or dark orange when you’re inactive, because when that happened to me I got really anxious I was like ‘Oh god I’m not doing well”‘*. Finally, two participants specifically noted that the feedback bar was distracting, directly working against the purpose of the virtual environment: *“The only thing that I had an issue with was that it was too big; it was just like right in my face. I would have preferred a small square in the corner or something. So that was where most of my attention went because I was so focused on like I really want it green; I want it green.”* (P16).

Participant **views on the accuracy of the feedback bar were diverse**. While some participants perceived it as reliable, others raised concerns about its performance, particularly during activities like reading. For instance, one participant who considered it quite reliable cited the way it functioned for them: *“I think it was pretty accurate, like a few times it would turn like yellow when I was like kind of just moving the mouse and not doing much. And then when I wasn’t doing anything it turned red and when I actually started doing homework and going forward and backward between PDFs and stuff it would turn green”* (P12). At the same time, several participants stated that the feedback was not accurate for them, especially for activities other than writing, and expressed how this in turn could lead to negative emotions: *“I wish it was more sensitive to tell if I was reading something. I like the idea of the bar, but if you’re doing readings or slides, or anything that doesn’t require immediate writing it would be distracting. When it turned red I would feel bad, but other than that I wouldn’t let it get to me.”* (P8).

## 5 DISCUSSION

We conducted a mixed-methods study to examine the use of VR technology for university students diagnosed with ADHD. In this study, the VR environment, together with the noise-canceling headphones, led to significant improvements in the concentration, effort/efficiency, and motivation of students with ADHD completing real-world academic tasks **(RQ1)**. The interviews with the participants yielded further insights into the experiences, impact, and value of VR for students with ADHD. Many participants expressed appreciation for the ability of VR to mitigate external and personal (e.g., phone) distractions, creating a more conducive environment for learning. Overall, these results are in line with previous research that found an increase in intrinsic motivation, concentration, and flow in knowledge workers working in VR [39, 40, 61], while extending it from pre-defined tasks to real-world tasks and providing evidence for the potential of VR for a population that could particularly benefit from this support: students with ADHD. By conducting our study in a VR environment representing a minimally furnished office space, we provide further evidence for the benefits of such an environment [39], yet more work focusing on VR environments for users with ADHD is required.

With respect to longer-term usage **(RQ2)**, more than two-thirds of the participants indicated that if VR headsets were available in the university library, they would use them to study and complete homework. The continuous high levels of concentration across up to 12 sessions spanning multiple weeks further support the value of a longer-term deployment and usage of VR. Although the majority of participants reported positive outcomes, it is important to note that students encountered some physical discomfort wearing the headsets. While some of the challenges are similar to those reported by Biener et al. for their week-long study [14], participants did not report simulator sickness or below-average usability. Partially, this may be attributed to the use of a high-quality VR headset rather than a simple and cheap model, but also due to the limited session duration of 50 minutes. The interview feedback suggests that 50 minutes can be a good duration for working on academic tasks in VR, yet it varies by individual and the experienced comfort.

Throughout the course of the experiment, we automatically monitored participants’ computer interactions using keyboard and mouse data. In later sessions, we used the data to provide participants with frequent and consistent performance feedback which took the form of a bar they could see in the VR environment. Overall, participants’ views on the feedback bar varied, and we did not find significant differences when comparing sessions with and without it with respect to motivation, effort, or concentration **(RQ3)**.

Our results highlight the need for further refinement to cater to individual preferences and needs. Future studies are needed to refine the VR system, addressing comfort issues, enhancing user experience, and improving the feasibility of students using VR without research support (e.g., help with setting up the headsets). Additionally, larger sample sizes will be needed to garner a comprehensive understanding (e.g., mediators and moderators) of the potential benefits and limitations of VR technology in educational contexts.

### 5.1 Do Students with ADHD Benefit from Receiving Performance Feedback?

To mimic continuous attention monitoring, we introduced a feedback bar into the VR environment that changed color based on the user’s interaction in the environment. For the subset of participants that took part in the sessions with the bar (sessions 6 to 10), we observed no significant differences in the sessions with and without the bar. This might be because simply being in VR already had such a strong impact on concentration (i.e., a floor effect). The mean concentration score in the first session was 3.78 (a score of 0 would represent a complete absence of difficulties with concentration in a 50-minute study period). Our qualitative findings further revealed a diverse range of participant responses to receiving feedback about their level of concentration within VR. While some participants expressed appreciation for its presence, others found the bar somewhat distracting.

Initially, the goal of the feedback bar was to alert participants whenever they were spending longer periods of time off task and get them back to their study materials. However, participants using VR had a remarkably low rate of distraction without receiving feedback. These findings, on the surface, suggest we may not need to give students performance feedback. Yet, experience tells us that students are likely to become distracted when working in a digital environment, even without ADHD [24]. So, it is important to note how the structure of our study may have added to this lack of distractions, and how real-world settings might lead to more temptations. In our study, participants worked in a lab setting for only 50 minutes and scheduled the sessions. If participants use VR in a dorm setting by themselves, they may be more likely to get off task. Furthermore, if they use the VR for longer sessions, they naturally have more chances to get distracted. Finally, if participants use VR as part of their daily routine (e.g., for an entire academic year), the novelty of using the headset will wane, and they are likely to find distractions on the internet, just as it happens in non-VR environments.

Another issue impacting conclusions on the feedback mechanism was that it may not be accurate or representative enough, which potentially shaped the relatively neutral responses from participants. In an attempt to examine our approach’s accuracy in detecting when participants lose focus inside the VR, we asked participants after each session to report the times they lost focus. According to participants, they never really lost focus in any of their sessions, which is strong support for the value of the VR but renders the evaluation infeasible on that level of granularity. For a more coarse-grained analysis, we performed a regression analysis with the concentration score for a VR session as the dependent variable and the average input level for the same session as the independent variable. The analysis revealed a statistically significant yet weak relationship between input level and concentration (slope=−0.52, intercept=5.15, *R*^2^=0.033, p<0.02). While these results indicate that there is a small relationship, they also show that there is a lot of space for improvement and that more data is needed to track a user’s attention on a per-session granularity. If better assessments of attention are developed, building, for example, on top of the keyboard and mouse data, participants may respond more positively to the overall feedback system. For example, to improve the accuracy of the attention detection algorithm, eye gaze tracking technology could be incorporated along with real-time analysis of screenshots, and even an automated detection of off-task websites (e.g., gaming, social, or sports sites). If participants noticed that the feedback was highly accurate and timely they may appreciate it more.

Finally, as pointed out by several participants, it is important in this context to carefully design how the feedback is provided since it can greatly impact how the user perceives it. For example, if the bar is *“too big”* (P16), it can distract people from their work, counteracting one of the main purposes of the VR environment. Similarly, the red color can be perceived as too intimidating and cause anxiety rather than motivate students to work, especially if there is no further explanation. More actionable feedback in the form of suggestions of what the student could do to get back to green could be valuable as P5 pointed out: *“the bar is a little intimidating ‘why is it going lower? …go back up!’ is what I would be thinking. Maybe instead of a bar, it could tell you ‘you have not been working as hard as you have been’ and give a suggestion.”*

### 5.2 How much Customization is Necessary?

While we only presented users with a single working environment (a cabin-type room with a desk), qualitative analyses revealed that most participants felt the environment was sufficient and did not suggest customization. Indeed, most requests for customization were around audio. Mirroring the divergence seen in previous studies [28], around half of the participants enjoyed the background noise (sounds of nature and birds chirping) and half found it annoying or distracting. We also had a few requests to enable listening to music while completing work. Future research will need to assess the impact of allowing music and perhaps the impact of the type of music on academic work completion [23].

While auditory customization was the most pressing issue for users, we hypothesize that offering general customization and variety in the VR environments may serve as a way to motivate students, particularly if VR technology is used with younger populations (e.g., middle school students with ADHD). For example, participants could earn points for staying on-task during VR sessions and those points could be used to unlock new environments such as a beach or a forest. This gamification system mimics what is often implemented with behavioral ADHD treatments [70]. Allowing participants to earn points and unlock new environments could help with motivation when using VR for a full academic semester.

### 5.3 How can we Deploy this Solution?

If VR technology for completing academic work is found to be effective in larger randomized trials, it will be important to consider how to scale up availability at schools and universities. Interestingly, our qualitative analyses revealed that college students with ADHD felt that the system would not be as effective if it was available in their homes/dorms. Participants noted that if VR was available in their home/dorm, they would be more likely to get distracted, also using the novel hardware to watch 3D movies and play games. This concern would be less of an issue if schools adopted VR strictly for testing situations. For example, VR may have significant benefits for secondary schools completing high-stakes standardized testing as an alternative to a private room. Interestingly, our qualitative analyses revealed that 76% of the interview participants indicated that they would use VR to complete work and study if it were available in the university library. Similar to how computers are handled at public libraries, VR headsets could have time limits, encouraging students to quickly get to work, and could block certain websites and types of use. Having VR headsets in university libraries would also be important to address issues surrounding equity in access to this technology.

### 5.4 Threats and Limitations

It is important to note that our ability to explore the impact of providing feedback about attention to participants was significantly limited because only 16 participants chose to attend the later sessions when this was evaluated. Specifically, while the N was 27 for evaluating the use of VR without feedback, due to attrition, the N dropped to 16 for evaluating the impact of providing feedback. As such, firm conclusions cannot be drawn from the analyses focused on using the visual bar to provide feedback and research with larger samples is needed. However, it is worth noting that our statistical analysis revealed no significant differences in concentration, effort/efficiency, motivation, or usability between participants who completed all 12 sessions and those who participated in fewer sessions and the M(SD) are remarkably similar. It is also important to note that the sample is largely comprised of emerging adults with ADHD Predominately Inattentive Presentation. While this is consistent with the literature on developmental trajectories of ADHD symptoms, the sample size is not large enough to evaluate whether VR is beneficial for college students with clinically significant levels of hyperactive/impulsive symptoms in addition to inattention symptoms. Future research is needed to evaluate the impact of ADHD presentation on the efficacy of VR.

Another important limitation to note is that participants were completing work and studying in a lab setting and a research assistant was always present. We felt that this was important for the initial test of VR, to assist with set-up and to be present if participants reported any side effects. Accordingly, it is not possible to fully parse out the effects of coming to the lab to complete work from the impact of using VR. That said, nearly all interviewees explicitly named the VR system and the removal of distractions in particular as a reason for their improved concentration, motivation, and effort. To gain a better understanding of the effects of VR, future research should utilize control groups, also examining control groups where the VR headset will be used in pass-through mode.

## 6 CONCLUSION

University students with ADHD often experience significant academic impairment due to challenges with distractibility and focus while studying and completing school work. In a study with 27 university students with ADHD, we evaluated the use of VR for completing real-world academic tasks and examined whether studying and completing homework in VR led to improvements in participant concentration, effort/efficiency, and motivation. Quantitative and qualitative data indicate that VR effectively reduced external and personal distractions and significantly enhanced concentration.

As part of this work, we implemented an automated feedback mechanism as a technology probe. The mixed gathered insights from participants suggest that, while automated feedback might provide some value to help students stay focused during academic tasks, two key areas require further attention: (1) creating an accurate and timely algorithm that can detect when a student is getting distracted; and (2) refining the feedback mechanism to match the effectiveness of real-world ADHD interventions, all while ensuring that the feedback itself does not become a source of distraction.

Our study also sheds initial light on an ideal duration for VR sessions and how and where to deploy VR headsets. Yet more research is needed in this direction, especially since they can have a large impact on the overall effectiveness of the approach. Further, it will be important to develop brief videos and manuals in the future that allow VR headsets to be set up, used, and adjusted during academic tasks without the involvement of research staff. Overall, this study suggests that VR has considerable promise for addressing distractibility and concentration difficulties in students with ADHD, which may lead to improved academic outcomes.

## Figures and Tables

**Table 1:** Mean and standard deviation for levels of concentration, effort/efficiency, and motivation when completing homework and studying (± indicates the standard deviation).

	Baseline	VR			
		All Sessions	1^*st*^ Session	With feedback	W/O feedback
Concentration	15.63 (±5.20)	3.73 (±3.12)	3.78 (±2.52)	3.85 (±3.83)	3.65 (±2.63)
Motivation	18.96 (±3.99)	23.72 (±4.13)	23.81 (±3.75)	23.58 (±4.73)	23.80 (±3.75)
Effort/Efficiency	15.15 (±4.39)	22.48 (±4.60)	21.44 (±3.77)	22.62 (±5.11)	22.40 (±4.29)

## CCS CONCEPTS

- **Human-centered computing** → **Empirical studies in HCI**; **Virtual reality**.

## Footnotes

1
https://jobsimulatorgame.com/

2 Note that the focus was to explore the emulation of the monitoring of attention levels and automatic feedback rather than determining an accurate proxy of attention levels.
